# Decreased brain and muscle ARNT-like protein 1 expression mediated the contribution of hyperandrogenism to insulin resistance in polycystic ovary syndrome

**DOI:** 10.1186/s12958-020-00592-1

**Published:** 2020-04-25

**Authors:** Junyu Zhai, Shang Li, Min Hu, Fangfang Di, Jiansheng Liu, Yanzhi Du

**Affiliations:** 1 Center for Reproductive Medicine, Ren Ji Hospital, School of Medicine, Shanghai Jiao Tong University, 845 Lingshan Road, Shanghai, 200135 China; 2 Shanghai Key Laboratory for Assisted Reproduction and Reproductive Genetics, Shanghai, 200135 China; 3 Community Health Service Center, Tianmu West Road, Jingan District, Shanghai, 200041 China

**Keywords:** BMAL1, Hyperandrogenism, Insulin resistance, PCOS, Circadian clock

## Abstract

**Background:**

The interface between environmental risk factors and genetic factors could contribute to the pathogenesis of hyperandrogenism and insulin resistance in polycystic ovary syndrome (PCOS); however, the underlying complex mechanism remains to be elucidated.

**Methods:**

We used dehydroepiandrosterone (DHEA)-induced PCOS-like rat model to measure circadian clock genes and insulin resistance-related genes. Additionally, we performed in vitro experiments in mature adipocytes to verify the molecular mechanisms.

**Results:**

DHEA-induced PCOS-like rats exhibited insulin resistance and arrhythmic expression of circadian clock genes in the liver and adipose tissues, particularly showing decreased brain and muscle ARNT-like protein 1 (BMAL1) expression. In addition, hyperandrogenism gave rise to negative regulation of BMAL1 expression to nicotinamide phosphoribosyltransferase and sirtuin 1, which further inhibited downstream glucose transporter type 4, leading to insulin resistance in mature adipocytes, which was consistent with our previous results in HepG2 cells.

**Conclusions:**

Decreased BMAL1 expression in the liver and adipose played a potentially novel role in the contribution of hyperandrogenism to insulin resistance, which might be a possible mechanism accounting for the pathogenesis of PCOS.

## Background

Polycystic ovary syndrome (PCOS) is a heterogeneous disorder characterized by hyperandrogenism, irregular menstrual cycle, and polycystic ovaries [1]. It is the most common endocrine disease affecting 6 –20% of women of reproductive age, with a varied pathogenesis ranging from genetic to environmental factors. The association between hyperandrogenism and insulin resistance leads to the pathogenesis of PCOS; however, the specific mechanism underlying the contribution of hyperandrogenism to insulin resistance is still not fully understood.

Insulin resistance, the state in which more insulin is required to maintain glucose homeostasis, is a common phenotype of PCOS patients and contributes to the pathogenesis of PCOS. Approximately 85% of PCOS patients, particularly those who are overweight, have insulin resistance [2]. Insulin signaling is complicated, and any changes in the process, such as phosphorylation of insulin receptors, insulin receptor substrate-1, or the phosphoinositide-3-kinase (PI3K)/protein kinase B (AKT) pathway and the subsequent expression of glucose transporter 4 (GLUT4), may play a role in the insulin-resistant state of PCOS [3]. Studies have indicated that multiple environmental risk factors such as continuous darkness or illumination, excessive food intake, lack of *exercise*, and environmental endocrine disruptors could contribute to the onset of PCOS [4].

Circadian clock is the biochemical oscillator that synchronizes human activities with solar time, and this internally synchronized circadian clock enables humans to coordinate their biology and behavior with daily environmental changes corresponding to the day–night cycle. The circadian clock of each tissue synchronizes endogenous and exogenous signals to regulate the transcriptional activity throughout the day in a tissue-specific manner through the regulation of circadian clock genes [5]. Circadian locomotor output cycles kaput (CLOCK) and brain and muscle ARNT-like protein 1 (BMAL1) heterodimers bind to cis-acting E-box elements to drive the transcription of cryptochrome (*CRY*) and period (*PER*), which subsequently return to the nucleus and repress their own transcription [6].

Circadian misalignment, a typical feature of jet lag and shift work, induces insulin resistance in humans [7], and elevates serum glucose and insulin levels in rats [8]. Mutations of the core circadian clock genes have been linked to the characteristics of the metabolic syndrome commonly associated with PCOS [9]. Our previous work has also presented that the arrhythmic expressions of circadian clock genes due to constant darkness result in the metabolic and reproductive hallmarks of PCOS in rats [10]. Reportedly, sleep disturbances are doubled in PCOS patients, and obstructive sleep apnea is a common feature of these patients. An appropriately classical pharmacological treatment combined with melatonin is recommended for PCOS patients to restore their endocrine–metabolic and reproductive functions [11]. More importantly, hyperandrogenism, the primary PCOS inducer, disrupts circadian organization in female rats [12] and female mice [13]. Although morning circadian misalignment is suggested associated with worse insulin sensitivity and higher serum free testosterone levels in girls with PCOS and obesity [14], the association among hyperandrogenism, metabolic homeostasis, and circadian clock function at the cellular level remains poorly understood.

In this study, we aimed to investigate the role of circadian clock genes in the contribution of hyperandrogenism to insulin resistance, which might also aid in determining appropriate interventions for PCOS patients.

## Materials and methods

### PCOS-like rat model

Female Sprague–Dawley (SD) rats (3 weeks old) were divided into five rats per cage under constant environmental conditions with a 12-h light–dark cycle. Food and water were provided ad libitum. The female rats in the dehydroepiandrosterone (DHEA) group (*N* = 30) received daily injections (s.c.) of DHEA (Langchem, Shanghai, China) (6 mg/100 g body weight) for 4 weeks, starting at the age of 3 weeks. The control group (*N* = 30) was injected the same volume of peanut oil. The PCOS-like rat model was selected according to previously reported criteria including estrous cycle, glucose tolerance test (GTT), and hematoxylin and eosin (HE) staining of ovarian tissues [3]. The body weight was detected every week from 0 to 4 weeks during the model building. After 4-week treatment, six rats from each group were killed every 5 h from ZT0 to ZT20. ZT0 was defined as 7:30 a.m. according to the lights-on time of the animal laboratory. Adipose, liver, ovaries, and serum were collected immediately after decapitation and stored for further analyses.

### GTT

After the 4-week treatment, female rats in the control and DHEA groups were kept under fasting for 16 h (5 p.m. to 9 a.m.) with free access to drinking water. Then, D-glucose (2.0 g/kg body weight) was intraperitoneally injected into each rat. Blood glucose levels were measured before and 30, 60, 90, and 120 min after the D-glucose injection using an Accu-Chek glucose monitor (Roche Diagnostics Corp., Basel, Switzerland).

### HE staining

Rat ovaries were fixed with 4% paraformaldehyde and embedded in paraffin. Tissue sections (thickness, 5 μm) were prepared, followed by deparaffinization and rehydration through a graded ethanol series. Sections then were stained in hematoxylin for 5 min and differentiated by hydrochloric acid for 30 s. Finally the sections were incubated in eosin for 2 min before covering the slide and visualizing under a microscope (Zeiss, Oberkochen, Germany).

### Cell culture

3T3-L1 preadipocytes were cultured in Dulbecco’s modified eagle medium (DMEM)/high glucose (Gibco, Grand Island, NY, USA) supplemented with 10% fetal bovine serum (FBS) (Gibco) and 1% penicillin–streptomycin–neomycin (Gibco) at 37 °C in a humidified atmosphere with 5% CO_2_. 3T3-L1 preadipocytes were induced to differentiate into mature adipocytes as previously described [15]. Oil red O staining (Sigma Chemical, St. Louis, MO, USA) was used to identify the mature adipocytes.

### Small interfering (si) RNA knockdown

A mixture of siRNA (50 pmol) and RNAiMAX (Invitrogen, Carlsbad, CA, USA) (9 μl) in OPTI-MEM (250 μl) was added to each well, and target genes were detected after 48 h. The specific sequences of the targeted genes were as follows:

- *Bmal1* siRNA, 5′- CCACCAACCCAUACACAGAAGCAAA − 3′,
- *Nampt* siRNA, 5′- GGCACCACUAAUCAUCAGATT − 3′,
- *Sirt1* siRNA, 5′- TTAGTGAGGAGTCCATCGG − 3′,
- Scrambled siRNA (NC), 5'-CCACCAAAUACACACGAAGCCCAAA-3'.

### Glucose uptake

Glucose uptake in cell lines was measured after insulin stimulation (100 nM for 20 min) using a Glucose Uptake-Glo™ Assay (Promega, Wisconsin, USA) according to manufacturer’s instructions.

### Enzyme-linked immunosorbent assay (ELISA)

The rats serum were detected using ELISA kits for mouse/rat leptin quanticine (R&D Systems, MN, USA), rat total adiponectin/Acrp30 quantikine (R&D), testosterone (Cayman Chemical, Michigan, USA), rat luteinizing hormone (LH) (MyBiosource, San Diego, USA), and rat follicle-stimulating hormone (FSH) (Biomatik, Ontario, Canada). Nicotinamide adenine dinucleotide (NAD^+^) levels in mature adipocytes were determined using an NAD/NADH Assay Kit (Abcam, Cambridge, UK). All procedures were performed according to manufacturer’s instructions.

### Western blot

Approximately 40 μg of protein was separated on a 10% SDS gel, following which it was transferred to a nitrocellulose membrane. The nonspecific binding sites of the nitrocellulose membrane were blocked using 5% nonfat milk and then incubated with anti-BMAL1 (Abcam) (1:1000), anti-T-AKT (Cell Signaling Technology, Danvers, MA, USA) (1:1000), anti-P-AKT (Ser473) (Cell Signaling Technology) (1:1000), and anti-GAPDH (Abcam) (1:5000) antibodies at 4 °C overnight. After washing, the blot was incubated and diluted with the corresponding peroxidase-conjugated secondary antibodies for 1 h at room temperature. Finally, the protein signals were detected using the enhanced chemiluminescent detection system (Millipore, Billerica, MA), and the ratio of a target protein to that of the intensity of GAPDH was obtained as each target protein level.

### Real-time quantitative polymerase chain reaction (RT-qPCR)

Total RNA was extracted from cells and rat tissues using an animal total RNA isolation kit (FOREGENE, Chengdu, China) and then reverse transcribed into cDNA (TAKARA, Dalian, China). RT-qPCR was used to detect the abundance of target genes. Following this, the results were analyzed through the ΔΔCt method using *β-Actin* as the housekeeping gene. The primer sequences targeted genes are presented in Table 1.

**Table 1:** The primer sequences of the RT-qPCR tested genes

Gene	Primer forward (5′ to 3′)	Primer reverse (5′ to 3′)
*Bmal1* (rat)	GGCTGTTCAGCACATGAAAAC	GCTGCCCTGAGAATTAGGTGTT
*Clock* (rat)	CTTCCTGGTAACGCGAGAAAG	GTCGAATCTCACTAGCATCTGAC
*Per1* (rat)	GATGTGGGTGTCTTCTATGGC	AGGACCTCCTCTGATTCGGC
*Per2* (rat)	CAGGTTGAGGGCATTACCTCC	AGGCGTCCTTCTTACAGTGAA
*Cry1 (rat)*	CACTGGTTCCGAAAGGGACTC	CTGAAGCAAAAATCGCCACCT
*Cry2 (rat)*	CACTGGTTCCGCAAAGGACTA	CCACGGGTCGAGGATGTAGA
*Sirt1 (rat)*	TGATTGGCACCGATCCTCG	CCACAGCGTCATATCATCCAG
*Nampt (rat)*	CCTGGTATCCAATTACAGTGGC	CCAAATGAGCAGATGCCCCTAT
*Glut4* (rat)	ACACTGGTCCTAGCTGTATTCT	CCAGCCACGTTGCATTGTA
*Pparg (rat)*	GGAAGACCACTCGCATTCCTT	GTAATCAGCAACCATTGGGTCA
*β-Actin* (rat)	GGCCAACCGTGAAAAGATGACC	AACCCTCATAGATGGGCACAG
*Bmal1 (mouse)*	ACAGTCAGATTGAAAAGAGGCG	GCCATCCTTAGCACGGTGAG
*Clock (mouse)*	ATGGTGTTTACCGTAAGCTGTAG	CTCGCGTTACCAGGAAGCAT
*Per1 (mouse)*	GAATTGGAGCATATCACATCCGA	CCCGAAACACATCCCGTTTG
*Per2 (mouse)*	CTCCAGCGGAAACGAGAACTG	TTGGCAGACTGCTCACTACTG
*Sirt1 (mouse)*	ATGACGCTGTGGCAGATTGTT	CCGCAAGGCGAGCATAGAT
*Nampt (mouse)*	GCAGAAGCCGAGTTCAACATC	TTTTCACGGCATTCAAAGTAGGA
*Glut4 (mouse)*	GGACCGGATTCCATCCCAC	TCCCAACCATTGAGAAATGATGC
*Pparg (mouse)*	CTCCAAGAATACCAAAGTGCGA	GCCTGATGCTTTATCCCCACA
*β-Actin (mouse)*	GTGACGTTGACATCCGTAAAGA	GCCGGACTCATCGTACTCC

### Statistical analysis

Statistical analyses were performed using the SPSS software package (version 22, SPSS Inc., Chicago, USA). For normally distributed data, we used paired Student’s t test or one-way analysis of variance followed by the Newman-Keuls multiple comparison test. For data not normally distributed, we applied the Kruskal-Wallis test followed by Dunn’s multiple comparison test. Data were expressed as mean ± SEM and significance was set at *p* < 0.05.

## Results

### DHEA treatment resulted in both reproductive and metabolic abnormalities in PCOS-like rats

**Fig. 1:**
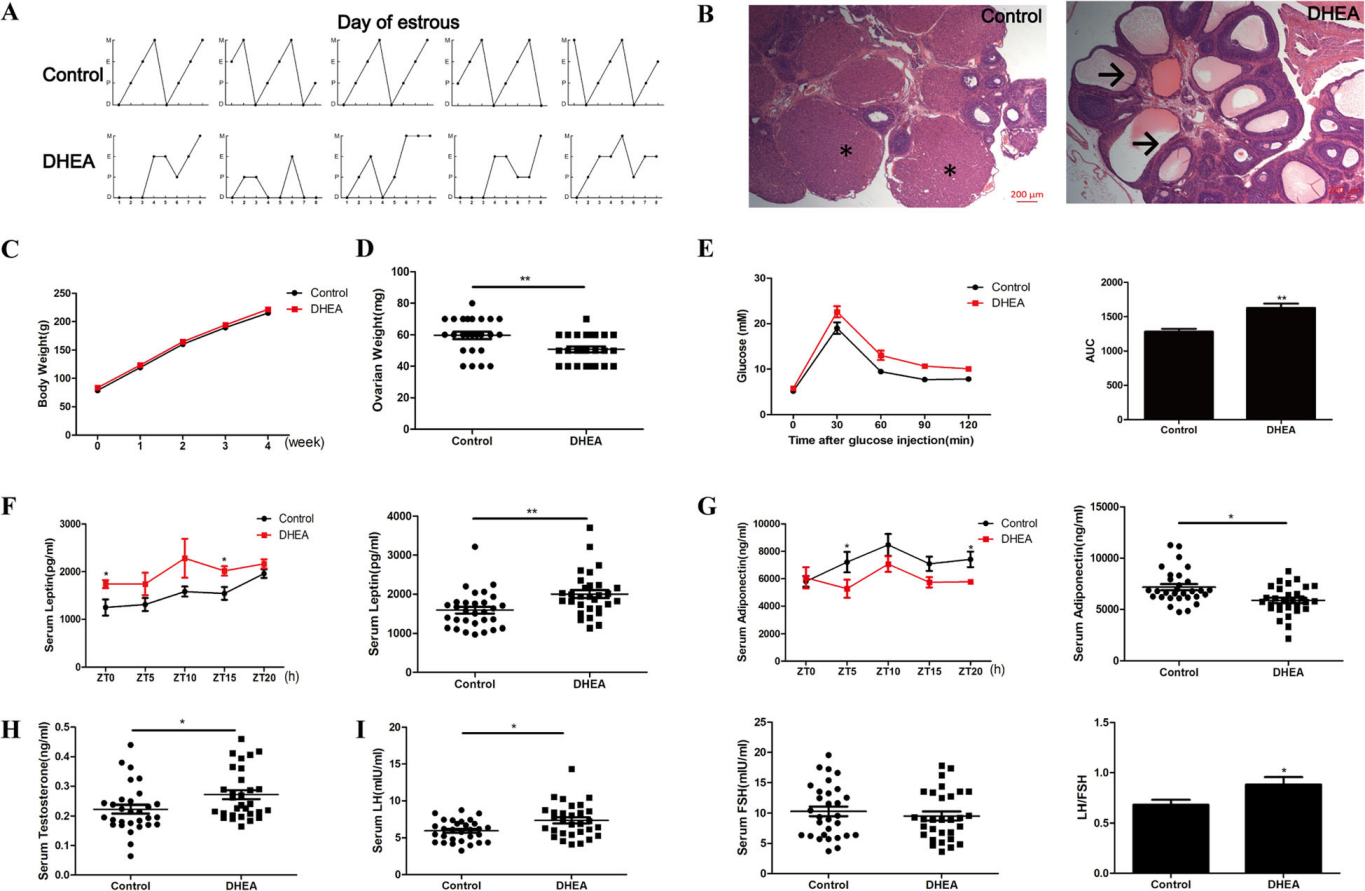
Characteristics of DHEA-induced PCOS-like rat model. **a** Estrous cycles of control and DHEA rats in the last 8 days. E (estrum), P (proestrus), M (metestrus), D (diestrus). **b** HE staining of the ovaries of control and DHEA rats. * for CL, → for cystic follicles. **c** Body weight of control and DHEA rats over 4 weeks. **d** Ovarian weight of control and DHEA rats after 4-week treatment. **e** GTT and area under the curve of control and DHEA rats after 4-week treatment. **f** Serum leptin levels in control and DHEA rats after 4-week treatment. **g** Serum adiponectin levels in control and DHEA rats after 4-week treatment. **h** Serum testosterone levels in control and DHEA rats after 4-week treatment. **i** Serum LH and FSH levels as well as LH/FSH ratio in control and DHEA rats after 4-week treatment. *N* = 30 per group. * *p* < 0.05, ** *p* < 0.01

To verify the potential relationship among hyperandrogenism, insulin resistance, and the circadian clock, DHEA was used as a trigger in female SD rats for 4 weeks. The DHEA group exhibited acyclicity, whereas the control group exhibited regular and complete estrous cycles in the last 8 days before decapitation (Fig. 1a). Moreover, the number of corpora lutea was decreased, whereas that of cystic follicles was increased in the ovaries of the DHEA group rats than in those of the control group rats (Fig. 1b). DHEA group rats showed a decreasing tendency in ovarian weight after 4-week treatment (Fig. 1d). However, no change was detected in the body weight between 2 groups during the whole process of rat model building (Fig. 1c). The higher area under the curve for GTT (Fig. 1e), increased serum leptin levels (Fig. 1f), and decreased serum adiponectin levels (Fig. 1g) after 4 weeks of DHEA treatment indicated impaired glucose metabolism and insulin sensitivity in DHEA group rats. Meanwhile, serum testosterone levels (Fig. 1h) as well as serum LH levels and LH/FSH ratio (Fig. 1i) were higher in this group. The combined findings of the examination of estrous cycle, ovarian histology, GTT, and serum testosterone and LH levels suggested that DHEA treatment induced a rat model with PCOS-like clinical symptoms, especially in a state of insulin resistance [16].

### Disrupted rhythmic expression of circadian clock genes and insulin resistance-related genes was detected in the liver tissue of PCOS-like rats

**Fig. 2:**
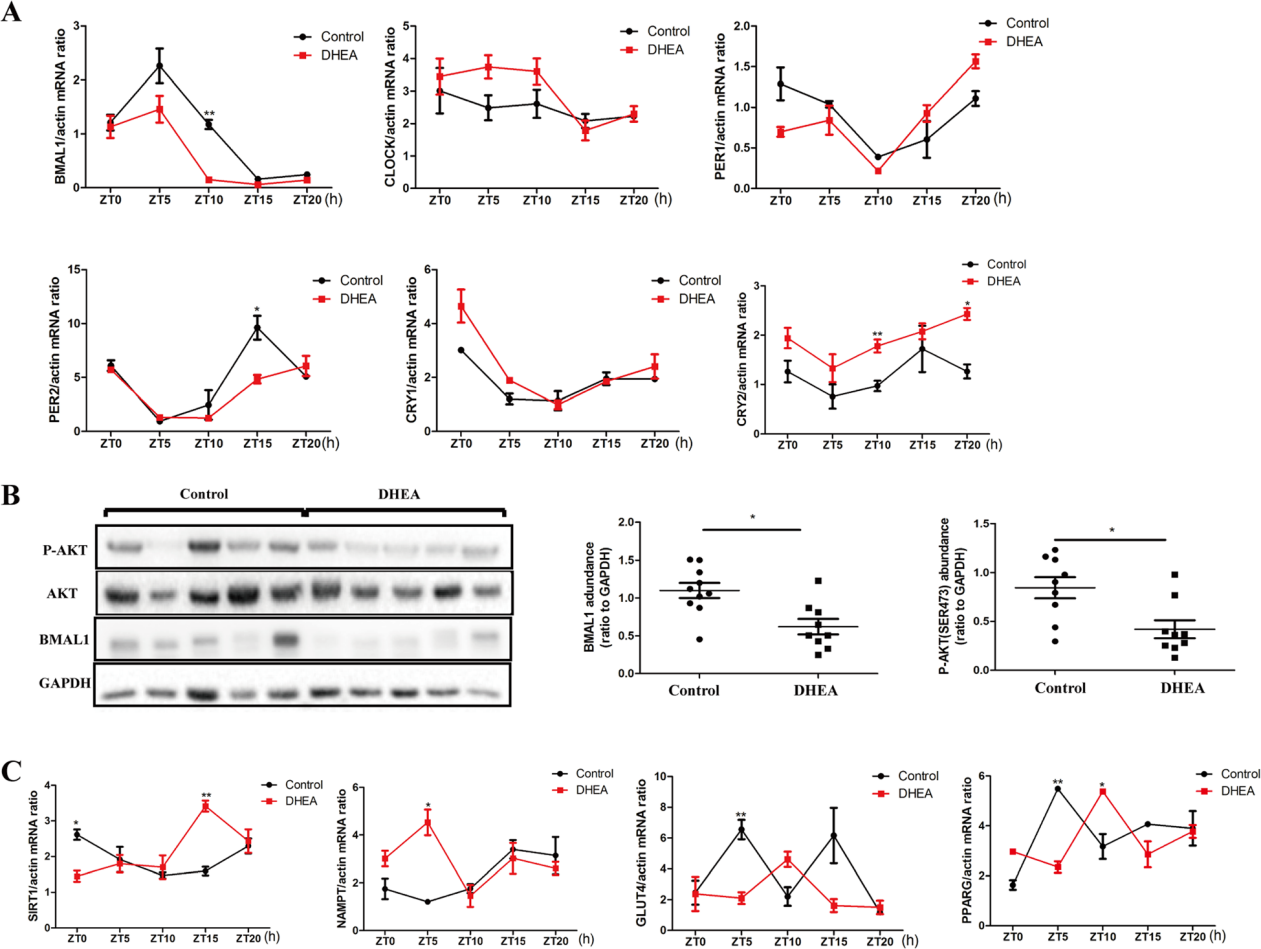
Expression of circadian clock genes and insulin pathway genes in the liver tissue of control and DHEA rats. **a***Bmal1, Clock, Per1, Per2, Cry1,* and *Cry2* mRNA expressions in the liver of control and DHEA rats. **b** P-AKT, T-AKT, and BMAL1 protein expressions in the liver of control and DHEA rats. Left, a representative western blot is shown. Middle, the immunoreactive bands for BMAL1 were quantified densitometrically. Right, the immunoreactive bands for AKT phosphorylation were quantified densitometrically. **c** mRNA expression of insulin pathway-associated genes including *Sirt1, Nampt, Glut4,* and *Pparg* in the liver of control and DHEA rats. *N* = 6 per time point and *N* = 30 for each group in total. * *p* < 0.05, ** *p* < 0.01

We investigated circadian genes and insulin pathways in the liver tissue of PCOS-like rats. *Sirtuin 1* (*Sirt1*) and *nicotinamide phosphoribosyltransferase* (*Nampt*) are the rhythmically expressed genes closely associated with insulin resistance [17, 18]. *Bmal1* mRNA expression decreased in the liver of PCOS-like rats compared with that of control rats, with the most significant reduction at ZT10. Moreover, *Per2* mRNA expression displayed a tendency to decrease at ZT15 and *Cry2* mRNA expression significantly increased at ZT10 and ZT20, whereas no difference was found in the expression of other circadian clock genes (Fig. 2a). The decreased BMAL1 and P-AKT protein expression in the liver implied the association between circadian clock genes and the insulin-resistant state in PCOS (Fig. 2b), which was further supported by the disrupted rhythmic mRNA expression of *Sirt1*, *Nampt*, *Glut4*, and *peroxisome proliferator activated receptor gamma (Pparg)*. Specifically, *Sirt1* mRNA expression in PCOS-like rats decreased at ZT0 and increased at ZT15, respectively. *Nampt* mRNA expression increased and *Glut4* mRNA expression decreased at ZT5. Furthermore, *Pparg* mRNA expression decreased at ZT5 and increased at ZT10, respectively (Fig. 2c).

### Disrupted rhythmic expression of circadian clock genes and insulin resistance-related genes was detected in the adipose tissue of PCOS-like rats

**Fig. 3:**
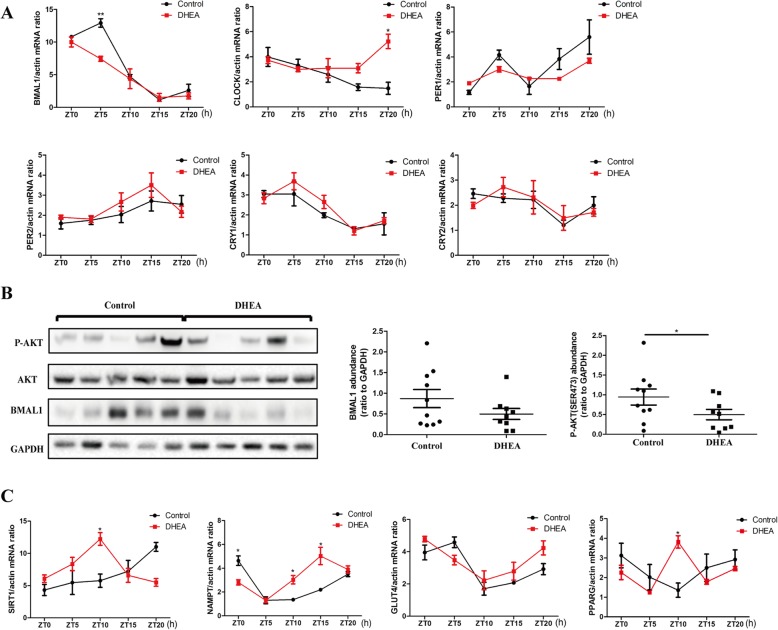
Expression of circadian clock genes and insulin pathway genes in the adipose tissue of control and DHEA rats. **a***Bmal1, Clock, Per1, Per2, Cry1,* and *Cry2* mRNA expressions in the adipose of control and DHEA rats. **b** P-AKT, T-AKT, and BMAL1 protein expressions in the adipose of control and DHEA rats. Left, a representative western blot is shown. Middle, the immunoreactive bands for BMAL1 were quantified densitometrically. Right, the immunoreactive bands for AKT phosphorylation were quantified densitometrically. **c** mRNA expression of insulin pathway-associated genes including *Sirt1, Nampt, Glut4,* and *Pparg* in the adipose of control and DHEA rats. *N* = 6 per time point and *N* = 30 for each group in total. * *p* < 0.05, ** *p* < 0.01

We also explored circadian gene expression in the adipose tissue of PCOS-like rats. *Bmal1* mRNA expression in PCOS-like rats significantly decreased at ZT5, whereas *Clock* mRNA expression increased at ZT20 (Fig. 3a). Adipose tissue of PCOS-like rats also showed a reduction in BMAL1 and P-AKT protein expression (Fig. 3b). A difference in the mRNA expression of *Sirt1*, *Nampt*, and *Pparg*, but not *Glut4*, in adipose tissue was discovered between PCOS-like rats and control rats. Specifically, *Nampt* mRNA expression in PCOS-like rats was decreased at ZT0 and increased at ZT10 and ZT15. Both *Sirt1* and *Pparg* mRNA expressions were increased at ZT10 (Fig. 3c).

### Hyperandrogenism facilitated BMAL1-mediated insulin resistance through the NAMPT/NAD^+^/SIRT1 pathway in mature adipocytes

**Fig. 4:**
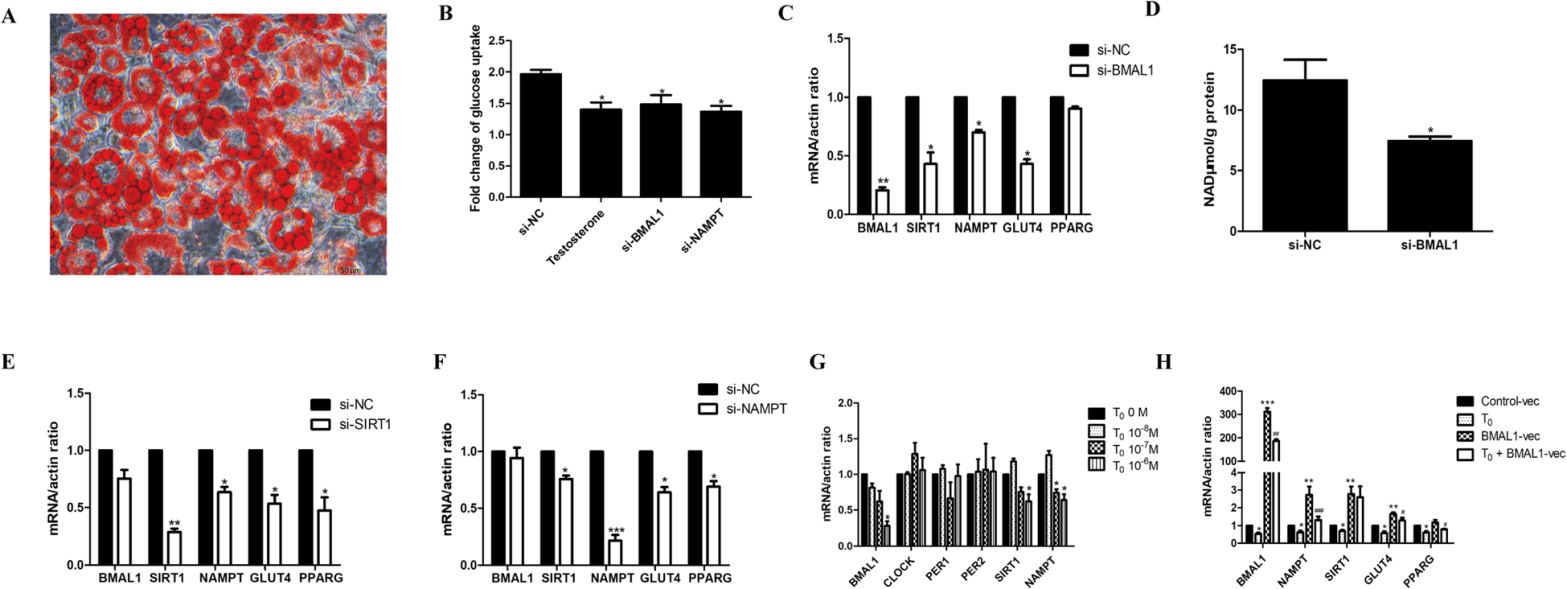
Hyperandrogenism facilitated BMAL1-mediated insulin resistance through the NAMPT/NAD^+^/SIRT1 pathway in mature adipocytes. **a** The mature adipose cells were differentiated from 3 T3-L1 preadipocytes after oil red O staining. **b** Glucose uptake after NC siRNA, testosterone (10^− 6^ M for 24 h), *Bmal1* siRNA, or *Nampt* siRNA treatment in mature adipocytes. Glucose uptake was measured after insulin stimulation (100 nM for 20 min). **c***Bmal1*, *Sirt1*, *Nampt*, *Glut4*, and *Pparg* mRNA expressions after knocking down *Bmal1* in mature adipocytes. **d** Cellular NAD^+^ level after knocking down *Bmal1* in mature adipocytes. **e***Bmal1*, *Sirt1*, *Nampt*, *Glut4*, and *Pparg* mRNA expressions after knocking down *Sirt1* in mature adipocytes. **f***Bmal1*, *Sirt1*, *Nampt*, *Glut4*, and *Pparg* mRNA expressions after knocking down *Nampt* in mature adipocytes. **g***Bmal1*, *Clock*, *Per1*, *Per2*, *Sirt1*, and *Nampt* mRNA expressions after treatment with different doses of testosterone for 24 h in mature adipocytes. **h***Bmal1*, *Nampt*, *Sirt1*, *Glut4*, and *Pparg* mRNA expressions after *Bmal1* overexpression and further incubation with testosterone (10^− 6^ M for 24 h) in mature adipocytes. * *P* < 0.05, ** *P* < 0.01, *** *P* < 0.001 against NC siRNA cells or against T_0_ 0 M cells or against Control-vec cells; # *P* < 0.05, ## *P* < 0.01, ### *P* < 0.001 against BMAL1-vec cells

In our previous work, we have certified that testosterone treatment decreases BMAL1 expression, and further reduces glucose uptake by inhibiting the NAMPT/NAD^+^/SIRT1 pathway and GLUT4 expression in HepG2 cells [10]. Mature adipocytes induced by the 3T3-L1 cell line are always used as the cell model for molecular research studies conferring to adipose tissue. Therefore we used this cell line to explore the mechanism of association between BMAL1 and insulin resistance in adipose tissues. Oil red O staining was used to identify the mature adipocytes (Fig. 4a).

We found that knocking down *Bmal1* or *Nampt* decreased glucose uptake in mature adipocytes (Fig. 4b). NAMPT-mediated NAD^+^ biosynthesis plays a critical role in numerous biological processes through the NAD^+^-dependent deacetylase SIRT1 [19]. Consequently, we further verified the regulation of BMAL1 and the NAMPT/NAD^+^/SIRT1 pathway. Knocking down *Bmal1* decreased *Nampt*, *Sirt1*, and *Glut4* mRNA expression as well as cellular NAD^+^ levels in mature adipocytes (Fig. 4c, d). Moreover, knocking down *Nampt* or *Sirt1* decreased *Glut4* mRNA expression without any effect on *Bmal1* expression (Fig. 4e, f). Therefore, *Bmal1* knockdown was suggested to reduce glucose uptake through the inhibition of the NAMPT/NAD^+^/SIRT1 pathway and *Glut4* expression in mature adipocytes.

Additionally, *Bmal1*, *Nampt*, and *Sirt1* mRNA expressions were decreased in a dose-dependent manner in mature adipocytes after testosterone treatment, whereas *Clock*, *Per1* and *Per2* mRNA expressions unchanged (Fig. 4g). Treatment with testosterone also decreased glucose uptake in mature adipocytes (Fig. 4b). Testosterone treatment partly hindered the increased NAMPT/NAD^+^/SIRT1 pathway and *Glut4* expression after *Bmal1* overexpression. Concurrently, compared with the control group, the addition of testosterone after *Bmal1* overexpression did not decrease the expression of downstream factors as testosterone treatment alone did (Fig. 4h). Thus, hyperandrogenism might facilitate insulin resistance via BMAL1-mediated pathway in mature adipocytes.

## Discussion

In this study, we demonstrated that DHEA-induced PCOS-like rats exhibited insulin resistance as well as arrhythmic expression of circadian clock genes in the liver and adipose, particularly showing decreased BMAL1 expression. Meanwhile, hyperandrogenism led to the negative regulation of BMAL1-induced expression of NAMPT/NAD^+^/SIRT1 pathway, further inhibiting downstream GLUT4 and contributing to insulin resistance in mature adipose cells, which was consistent with our previous results in HepG2 cells [10]. Thus, our study uncovered the potentially novel role of BMAL1 in the contribution of hyperandrogenism to insulin resistance in PCOS.

Environmental risk factors combined with genetic factors give rise to hyperandrogenism and insulin resistance, both of which are known to be involved in the onset of PCOS [20]. Circadian disruption, especially constant darkness, induces insulin resistance of PCOS in rats, including increased fasting blood glucose level and increased serum insulin and leptin levels [10]. To investigate the relationship between circadian dysfunction and glucose metabolism, we used liver and adipose tissues of the DHEA-induced PCOS-like rat model as these are the two principal tissues involved in insulin resistance. Rhythmic disorder in the expression of circadian genes, especially *Bmal1*, as well as insulin pathway genes was observed in both liver and adipose of PCOS-like rats. Our previous study has shown the decreased *BMAL1* mRNA expression in PCOS patients [10]. BMAL1 plays a vital role in lipid and glucose metabolism. It promotes de novo lipogenesis through insulin–mammalian target of rapamycin complex 2 (mTORC2)–AKT signaling in liver tissue [21], and *Bmal1*-null male and female mice have been reported to display increased adiposity [22]. *BMAL1* knockdown induces insulin resistance, as indicated by markedly impaired insulin-stimulated phosphorylation of insulin receptor and AKT pathway [18]. BMAL1 is also involved in estrogen synthesis via SIRT1 in PCOS patients [23]. Hence the change in BMAL1 expression plays a potential role in hyperandrogenism-induced insulin resistance in PCOS.

As a circadian upstream factor of SIRT1, NAMPT is involved in insulin resistance through PPARγ and adiponectin [17]. NAMPT and NAD^+^ display circadian oscillations, and the circadian transcription factor CLOCK upregulates NAMPT [17]. Furthermore, adipose-specific NAMPT is closely related to insulin resistance in the adipose tissue, liver, and skeletal muscle through the regulation of PPARγ and adiponectin [24]. Intelectin-1 promotes steroidogenesis in the granulosa cells of PCOS patients via NAMPT [25]. Previous study has confirmed the importance of the NAMPT/NAD^+^/SIRT1 pathway in the systemic regulation of glucose metabolism [26]. Based on the arrhythmic *Nampt* and *Sirt1* mRNA expressions in the liver and adipose of PCOS-like rats, we speculated that NAMPT and SIRT1 might be the bridge linking BMAL1 and insulin resistance in PCOS patients. Using mature adipocytes, we confirmed that hyperandrogenism inhibited the BMAL1-induced NAMPT/NAD^+^/SIRT1 pathway, and further reduced GLUT4 to suppress insulin sensitivity, which was consistent with our previous data in HepG2 cells (Figure S1) [10].

The effect of hyperandrogenism on circadian organization and clock function may be somewhat ubiquitous and represents a general impact of elevated androgen levels on circadian timing. Hyperandrogenism might affect the amplitude of clock output and the precision of clock control over behavior, but it might not affect circadian rhythms of clock gene expression in the suprachiasmatic nucleus [27]. Androgen receptor (AR) expression in suprachiasmatic nucleus neurons is not rhythmic, but it is significantly affected by circulating androgen levels. DHEA-induced growth suppression of preadipocytes is mediated via AR [28]. It is possible that AR expression in the liver and adipose tissues of PCOS-like rats might be differentially altered. Notably, AR expression in the liver and adipose tissues is closely associated with visceral fat mass and glucose homeostasis [29]. Thus, the association between AR signaling and BMAL1 in each tissue is also worth investigating in the future.

Among the limitations of this study is the fact that we did not detect the expression of circadian clock genes in the liver and adipose tissues of women with or without PCOS due to the scarcity of samples. Secondly, since we proposed and confirmed the role of BMAL1 in the contribution of hyperandrogenism to insulin resistance in PCOS, it might be a novel potential therapeutic target for PCOS patients. More experiments with feasible intervention such as melatonin treatment are urgently needed, and more experiments elaborating on the related molecular mechanisms will be required.

## Conclusions

We investigated the changes in circadian clock genes expression and their association with hyperandrogenism and insulin resistance, putting forward a new molecular mechanism to elaborate the contribution hyperandrogenism to insulin resistance in PCOS. Hyperandrogenism resulted in the negative regulation of BMAL1-induced expression of the NAMPT/NAD^+^/SIRT1 pathway, which further suppressed GLUT4, leading to insulin resistance in the liver and adipose tissues of rats. The disrupted rhythmic expression of circadian clock genes, specifically decreased BMAL1 expression, mediated the contribution of hyperandrogenism to insulin resistance in PCOS.

## Supplementary information

**Additional file 1 Figure S1.** Proposed signaling pathways underpinning the essential role of BMAL1 in the contribution of hyperandrogenism to insulin resistance in PCOS.

## Data Availability

The datasets generated during and/or analyzed during the current study are available from the corresponding author on reasonable request.

## Abbreviations

PCOS: Polycystic ovary syndrome
BMAL1: Brain and muscle ARNT-like protein 1
CLOCK: Circadian locomotor output cycles kaput
CRY: Cryptochrome
PER: Period
SD: Sprague–Dawley
DHEA: Dehydroepiandrosterone
GTT: Glucose tolerance test
HE: Hematoxylin and eosin
DMEM: Dulbecco’s modified eagle medium
FBS: Fetal bovine serum
ELISA: Enzyme-linked immunosorbent assay
LH: Luteinizing hormone
FSH: Follicle-stimulating hormone
RT-qPCR: Real-time quantitative polymerase chain reaction
NAD^+^: Nicotinamide adenine dinucleotide
NAMPT: Nicotinamide phosphoribosyltransferase
PPARG: Peroxisome proliferator activated receptor gamma
SIRT1: Sirtuin 1
GLUT4: Glucose transporter type 4
AR: Androgen receptor
