# Supplementary material for: Decreased brain and muscle ARNT-like protein 1 expression mediated the contribution of hyperandrogenism to insulin resistance in polycystic ovary syndrome
Source: Reprod Biol Endocrinol. 2020 Apr 25;18:32. doi: 10.1186/s12958-020-00592-1 (PMC7183135; doi:10.1186/s12958-020-00592-1)
Supplement: Supplementary file 1 — Additional file 1 Figure S1. Proposed signaling pathways underpinning the essential role of BMAL1 in the contribution of hyperandrogenism to insulin resistance in PCOS. [file 12958_2020_592_MOESM1_ESM.pdf]

Supplementary Figure 1

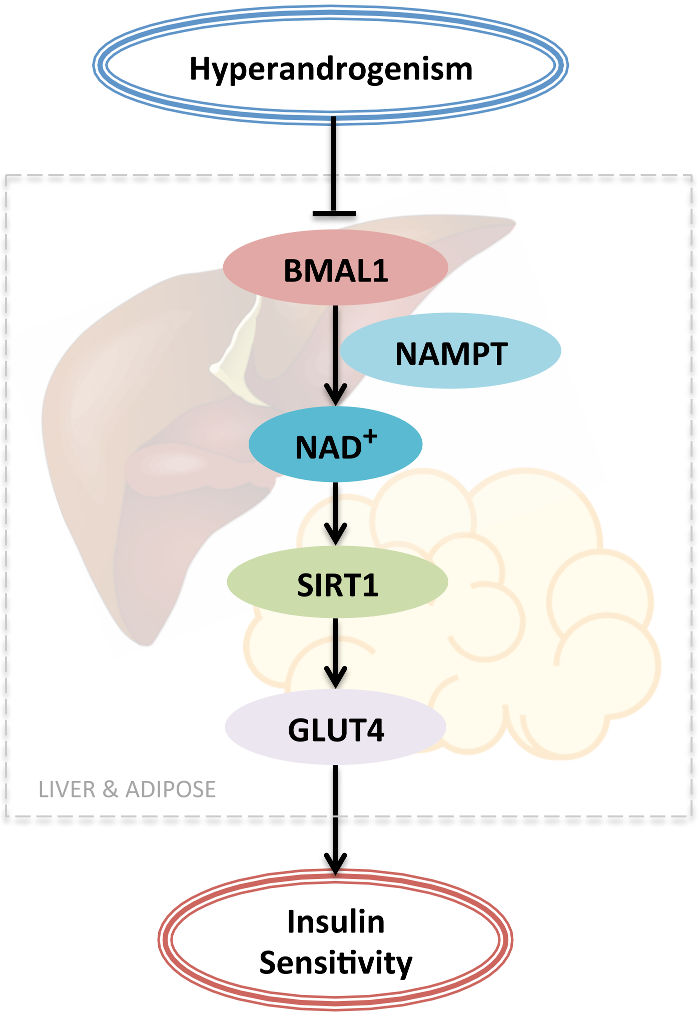

Figure S1. Proposed signaling pathways underpinning the essential role of BMAL1 in the contribution of hyperandrogenism to insulin resistance in PCOS.

Hyperandrogenism led to the negative regulation of BMAL1-induced expression of the NAMPT/NAD^+/SIRT1 pathway, which further inhibited downstream GLUT4, contributing to insulin resistance both in the liver and adipose tissue of PCOS.
